# Loss of the interaction between estradiol and insulin-like growth factor I in brain endothelial cells associates to changes in mood homeostasis during peri-menopause in mice

**DOI:** 10.18632/aging.101739

**Published:** 2019-01-11

**Authors:** Victor Munive, Jonathan A. Zegarra-Valdivia, Raquel Herrero-Labrador, Ana M. Fernandez, Ignacio Torres Aleman

**Affiliations:** 1 Cajal Institute, Madrid, Spain; 2 Ciberned, Madrid, Spain; 3 Universidad Nacional de San Agustín de Arequipa, Arequipa, Perú

**Keywords:** reproductive aging, mood, exercise, insulin-like growth factor 1, estrogen

## Abstract

We recently reported that exercise increases resilience to stress in young female mice. Underlying mechanisms include an interaction of the ovarian hormone estradiol (E2) with insulin-like growth factor I (IGF-I), and an increase in the hippocampal levels of the latter. Since changes in mood regulation during aging may contribute to increasing incidence of affective disorders at older age, we determined whether the protective actions of exercise are maintained at later ages. We found that during peri-menopause, exercise no longer improves resilience to stress and even becomes anxiogenic. Furthermore, the interaction seen in young females between the E2 α receptor (ERα) and the IGF-I receptor (IGF-IR) is lost at middle-age. In addition, E2 no longer induces IGF-I uptake by brain endothelial cells, and consequently, hippocampal IGF-I levels do not increase. Treatment of middle-aged females with an ERα agonist did not recover the positive actions of exercise. Collectively, these data indicate that the loss of action of exercise during peri-menopause may be related to a loss of the interaction of IGF-IR with ERα in brain endothelial cells that cannot be ameliorated by estrogen therapy. Changes in regulation of mood by physical activity may contribute to increased appearance of affective disorders along age.

## Introduction

Mood homeostasis varies throughout aging [1,2], but our knowledge of the underlying mechanisms remains incomplete. In females, an increased incidence of affective disorders already occurs at middle age [2]. At this time in life, female mice begin to experience ovarian cycle disturbances as they enter the peri-menopause, a transition period leading to reproductive senescence [3]. Accordingly, gonadal steroids such as estradiol (E2) start to change [4], and other hormones modulating reproduction, including those of the somatotropic axis (GH/IGF-I), also change [5,6].

We recently reported that in young female mice, exercise modulates resilience to stress through a concerted action of E2 with IGF-I in brain endothelial cells, a somewhat unexpected site of interaction for mood-regulatory mechanisms [7].

Since changes in estrogen activity has been suggested to underlie neurological changes during peri-menopause [8], we evaluated modulation of mood by exercise and analyzed the interactions between E2 and IGF-I in brain endothelial cells during this transitional period. We now report that the positive actions of exercise on resilience to stress are lost in female mice during peri-menopause. In parallel, the interaction of E2 and IGF-I in brain endothelial cells is also lost. Furthermore, estrogenic therapy did not rejuvenate the ability of exercise to modulate mood.

## RESULTS

### Modulation by exercise of stress resilience in middle age female mice

**Figure 1:**
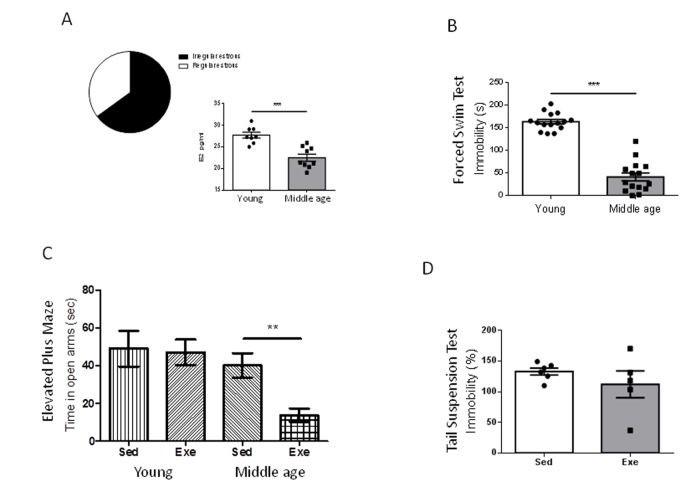
**Exercise actions in anxiety-like behavior and resilience to stress in middle-aged female mice at peri-menopause.** (**A**) Over 60% of middle age female mice were in constant estrous. E2 levels were significantly decreased in middle-age females as compared to young females. ***p<0.001 vs young (n=8-9). (**B**) Middle-aged female mice (9 months old) showed reduced immobility time in the forced swim test compared to young females (2 months old), indicating a lower “depressive-like” state (n=17 per group). (**C**) Anxiety-like levels measured in the elevated plus maze showed a profound anxiogenic effect of exercise in middle-aged female mice as compared to young mice (n= 5-10). Values of young females were taken from reference 7. (**D**) Exercise does not modify resilience to stress (measured by the tail suspension test delivered after the forced swim test) in middle-aged females (n=5-6). Exe: exercised mice; Sed: sedentary mice (in this and following figures).

We first confirmed that our middle-aged (9 months) female population was entering the reproductive senescence transition; over 60% of females already showed altered ovarian cycling -chronic diestrus (Figure 1A). Indeed, a 20% overall reduction in serum E2 as compared to young females, was found at this transitional period (Figure 1A, p<0.001 vs young females).

Middle-aged female mice show markedly reduced immobility time in the forced swim test compared to young females (2 months-old; p<0.001; Figure 1B). Conversely, performance in the elevated plus maze, or in the tail suspension test was similar in young and middle-aged females (Supplementary Figure 1A, B). However, middle-aged female mice did show a detrimental pattern of exercise modulation of these tests. Thus, while in young females exercise did not modify the time spent in the open arms in the elevated plus maze [7], in older females exercise reduced it (p<0.01 vs sedentary; Figure 1C). In addition, while in young females exercise robustly decreased the immobility time in the tail suspension test [7], in middle-aged females exercise did not modify it as compared to sedentary females (Figure 1D).

**Figure 2:**
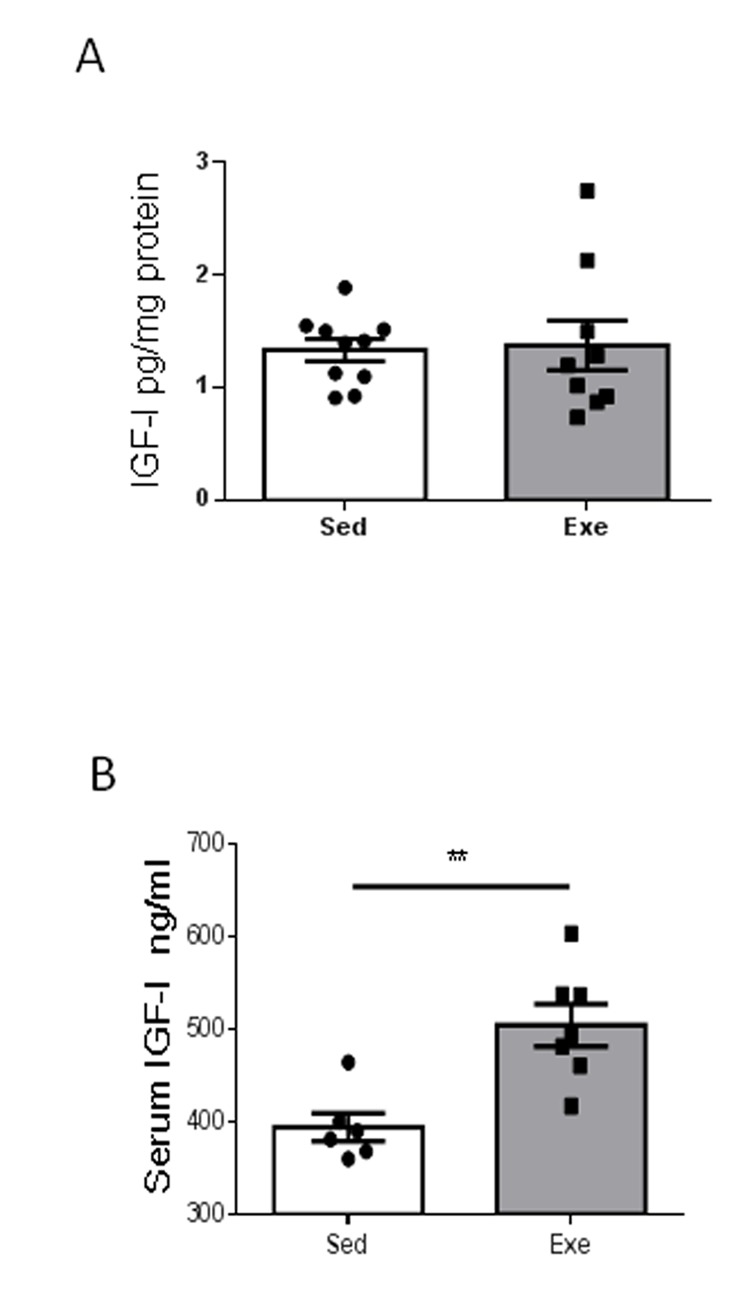
**Effects of exercise on IGF-I in middle-aged females.** (**A**) Hippocampal levels of IGF-I did not change after exercise in middle-aged female mice (n=9-10). (**B**) Serum IGF-I increased in middle-aged females after exercise (n= 6-7). **p<0.01 and ***p<0.001 vs respective control in this and following figures.

Since exercise regulation of resilience to stress in young females is accompanied by increased hippocampal IGF-I [7], we next determined whether middle-aged females show changes in hippocampal IGF-I after exercise, and found it to be unaltered (Figure 2A). However, exercise induced an increase in circulating IGF-I in middle-aged females (Figure 2B), while sedentary middle-aged females had a 22% decrease in serum IGF-I levels compared to sedentary younger ones (501 ± 29 ng/ml in young females vs 394 ± 37 ng/ml in middle-aged females; n=6-10, p<0.05).

**Figure 3:**
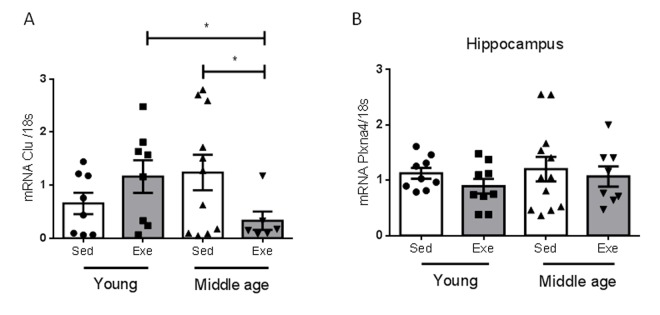
**Effects of exercise on clusterin and its receptor in middle-aged females.** (**A**) Hippocampal levels of clusterin (Clu) changed in an age-dependent manner in response to exercise (n=5-10, p<0.05). **(B**) Hippocampal levels of the Clu receptor Plxna4 did not change with age, nor with exercise (n=7-12). *p<0.05 vs respective control in this and following figures.

We determined whether the effects of aging on hippocampal responses to exercise are specific for IGF-I or also affect other proteins reportedly altered in the aging hippocampus during the reproductive senescence transition [9]. We chose two of them, and found that only one, clusterin, but not its receptor Plxna4, showed a distinct age-dependent pattern of responses to exercise (Figure 3).

### Estradiol no longer stimulates IGF-I uptake by brain endothelium at middle age

**Figure 4:**
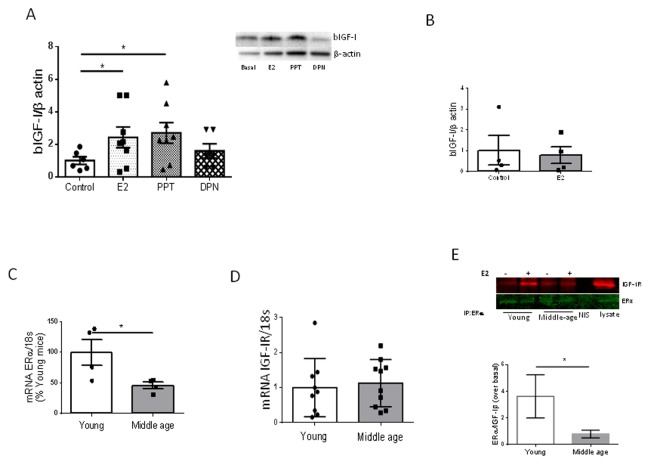
**Interactions between E2 and IGF-I.** (**A**) Uptake of biotinylated IGF-I (bIGF-I) by brain endothelial cells obtained from young female mice (2 months-old) is increased by estradiol (E2) acting through E2Rα. Note that only the ERα agonist PPT, but not the ERβ agonist DPN, mimics the actions of E2. Representative blots are shown at the right. β-actin was measured as a loading control (n=6). (**B**) Estradiol does not stimulate uptake of bIGF-I in brain endothelial cells obtained from middle-aged female mice (n= 4). (**C**) Levels of ERα mRNA were reduced in brain endothelia from middle-aged female mice (n=4). (**D**) Levels of IGF-IR mRNA remain unaltered in brain endothelia in middle-aged female mice compared to young mice (n=9-10). (**E**) Co-immunoprecipitation of ERα with IGF-IR showed a significantly decreased interaction in response to E2 in brain endothelial cells obtained from middle-aged female mice (n=4). Representative blot of an immunoprecipitation using anti-ERα is shown*.* NIS: non-immune serum. *p<0.05 vs respective control.

We previously observed that estradiol, acting through its alpha receptor (ERα) on brain vessels, is required to increase hippocampal IGF-I after exercise through increased uptake of IGF-I from the circulation [7]. Indeed, PPT, an ERα agonist, but not DPN, an ERβ agonist, mimicked the stimulatory action of E2 on IGF-I uptake by cultured brain endothelial cells from young female mice (Figure 4A). In contrast, E2 was no longer able to stimulate IGF-I uptake by brain endothelial cells of middle-aged females (Figure 4B). We examined levels of ERα in brain endothelium and found it to be decreased in 9 month old female mice as compared to young ones (Figure 4C). However, expression of IGF-I receptors (IGF-IR) in endothelial cells was unaltered in middle-aged females (Figure 4D). Because ERα and IGF-IR have been shown to interact in response to E2 [10], we next determined whether in middle-aged endothelium this interaction was altered as compared to young endothelium. Indeed, a significantly reduced interaction between these receptors was found (Figure 4E; p<0.05).

**Figure 5:**
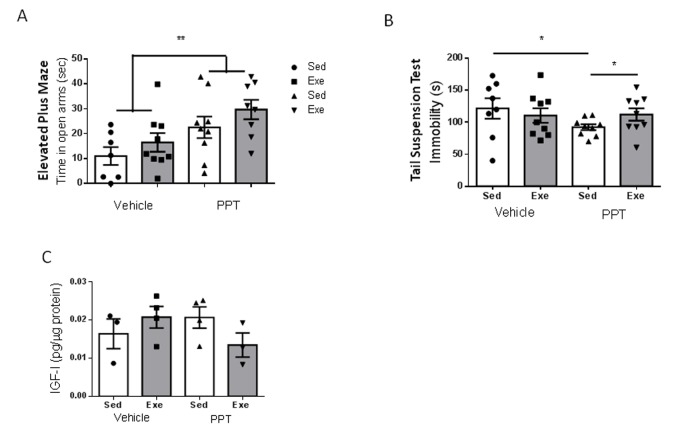
**Exercise modulation of anxiety and resilience to stress in middle-aged female mice after treatment with an ERα agonist.** (**A**) Administration of the ERα agonist PPT resulted in marked anxiolysis in middle-aged females, but exercise did not modify anxiety-like behavior, as measured in the EPM test (n=7-9). (**B**) PPT treatment increased resilience to stress, as indicated by reduced immobility in the tail suspension test, while exercise abrogated its effects (n=8-9). (**C**) PPT did not affect hippocampal IGF-I levels and did not significantly affect responses to exercise (n=3-4).

Since middle-aged females showed altered ovarian cycling, reduced ERα expression in brain endothelium, and reduced serum E2 levels (see above), we treated them with the ERα agonist PPT during 18 days. We aimed to increase estradiol signaling through this type of receptor because it is through this receptor subtype that E2 interacts with IGF-I. First of all, we observed that submitting middle-aged female mice to the stressful handling produced by daily ip injections of the vehicle was enough to reduce the time spent in the open arm of the elevated plus maze: from around 50 sec in un-injected controls (Figure 1C) to around 10 sec in vehicle-injected females (Figure 5A). However, PPT treatment partially restored the time spent in the open arms (p<0.01 vs sedentary vehicle; Figure 5A), and significantly decreased immobility in the tail suspension test (p<0.05 vs sedentary vehicle; Figure 5B). However, PPT treatment induced a distinct pattern of changes in the performance of behavioral tests after exercise, as compared to young females. Hence, slight, but not significant improvements were seen in the elevated plus maze after exercise, similarly to the effect of the vehicle (Figure 5A). On the other hand, PPT treatment resulted in increased immobility time in the tail suspension test (p<0.05 vs sedentary PPT; Figure 5B). In agreement with a lack of effect of E2 on IGF-I uptake by middle-aged endothelial cells (Figure 4B), all these pattern of changes were independent of hippocampal IGF-I levels, as neither PPT nor exercise affected them (Figure 5C).

## DISCUSSION

By using exercise as a physiological modulator of different mood traits in female mice, we now confirm and extend previous observations in male mice of relatively early changes in mood homeostasis during aging [11]; more specifically, between 2 (young) and 9 months (middle-aged). These changes take place in parallel with a loss of the interaction between IGF-I and estradiol at the blood-brain-barrier (BBB), as the latter no longer stimulates IGF-I uptake by brain endothelial cells in response to exercise. Altered responses to exercise at middle age were also seen in hippocampal levels of clusterin, a glycoprotein with wide actions including neuroprotection, that has been placed at the center of hippocampal gene changes during the reproductive senescence transition [9]. The latter suggests a broader pattern of changes in the hippocampus of middle-aged female mice in response to exercise.

Because IGF-I is a potent modulator of mood [12–15] and its intracerebroventricular administration has been shown to produce anxiolysis [16], the absence of a hippocampal IGF-I response to exercise probably contributes to the differences in the regulation by exercise of diverse mood traits observed in middle-aged females as compared to younger ones. These observations add further argument for uncoupling of estrogen function with other systems during reproductive senescence as an underlying process responsible for neurological changes during this period [8]. These findings in experimental animals may also bear relevance to the onset of mood alterations during peri-menopause described in women [17], pointing to BBB function as a possible area for further research.

However, while we do not know the mechanisms underlying the loss of interaction of estradiol with IGF-I during reproductive senescence in mice, the transition from fertility to infertility in women and female mice differs. Women slowly develop ovarian dysfunction with declining hormonal levels, whereas in female mice an initial persistent estrus with tonic E2 levels develops before E2 levels eventually decline. Since the timing of associated hormonal changes in the peri-menopause to menopause transition is not the same in humans and mice, it remains possible that the observed changes in mice develop as a consequence of chronic estrus, making the observed changes species-specific. Alternatively, similar changes may take place in humans but with a different timing. The latter is supported by known changes in mood during peri-menopause in women [18], but, at any rate, this warrants further study.

It is now widely accepted that estrogen replacement therapy in aging women is highly context dependent [19]. For instance, its late administration in aged women generally resulted in detrimental actions on brain function [20]. In our hands, treatment of middle-aged female mice with an ERα agonist had positive effects on various mood traits, confirming prior observations of E2 actions in aged female mice [21]. However, treatment with the ERα agonist did not restore the regulatory effects of exercise to pre-peri-menopause stages, nor did it restore exercise-induced increases in hippocampal IGF-I. We consider that the lack of effect of ERα agonist supplementation in these two latter parameters may be explained by two observations that are probably inter-connected: 1) lowered expression of ERα receptors in brain endothelium at middle age, which fits well with the observed reduction of this subtype of estrogen receptor in brain during aging through epigenetic processes [22], and 2) loss of the estradiol-induced interaction in middle-aged endothelia of ERα with IGF-IR [10].

Lack of increased IGF-I uptake in response to exercise is not due to changes in IGF-I receptor levels in middle-aged brain endothelium, as they remain unchanged. Moreover, serum IGF-I is increased after exercise in middle-aged females, which could be related to the reported decrease in basal levels of circulating IGF-I during age [23]. This increase after exercise differs from young females, which showed no changes in serum IGF-I after exercise [7]. Collectively, these observations suggest that the absence of increased IGF-I uptake by the brain is independent of the levels of circulating IGF-I or of its receptor in brain endothelium, and is probably related to an uncoupling with E2α signaling. Of note, increased brain uptake of serum IGF-I has been found to correlate with decreases in serum IGF-I [24], which further suggests that increased serum IGF-I levels after exercise in middle-aged females is due, at least in part, to low brain uptake.

In summary, changes in the regulation of anxiety-like behavior and resilience to stress in middle-aged female mice are associated with an absence of increased IGF-I in the brain, due to reduced ERα activity (lower expression of the receptor together with lower E2 levels) and decreased exercise-induced IGF-I uptake by brain endothelium. Whether interactions between E2 and IGF-I in other brain areas are also involved in the observed changes in the regulation of these mood traits cannot be discarded, as both ERα and IGF-IR have a profuse distribution in brain areas related to mood regulation [25–28]. Moreover, other age-related actions of E2 in the brain may be entirely independent of the presently observed interactions with IGF-I at the BBB [29].

## MATERIALS AND METHODS

### Animals and experimental procedures

Adult female C57BL/6J mice (2 and ∼9 months old; Harlan Laboratories, Spain) were housed in standard cages (48 × 26 cm^2^) with 5 animals per cage. Mice were kept in a room with controlled temperature (22°C) under a 12-12h light-dark cycle and fed with a pellet rodent diet and water *ad libitum*. All experimental protocols were performed during the light cycle. Animal procedures followed European guidelines (86**/**609**/**EEC and 2003**/**65**/**EC, European Council Directives) and were approved by the local Bioethics Committee (Government of the Community of Madrid). Thus, following the “reduction” guideline, repeating experiments in young animals was reduced as much as possible as these studies were previously performed by our group (same investigator handling the animals) and have been described elsewhere in detail [7]. We use the data of this recent publication as a reference throughout the manuscript.

Estrous cycles in female mice were monitored by daily inspection of vaginal smears. The material was collected at the same time each day during 10 days. Approximately 10 μl of 0.9% saline were gently flushed into the vagina with the tip of a plastic pipette three times, and the final flush placed onto a glass slide and observed under a light microscope with a 10× objective. Determination of the estrous cycle phase was based on published procedures [30] by examining the proportion of the following cell types: predominance of leukocytes (diestrous), predominance of nucleated epithelial cells (proestrous), predominance of cornified epithelial cells (estrous), and a mix of cell types with a predominance of leukocytes and a few nucleated epithelial and/or cornified squamous epithelial cells (metestrus). The E_2_ receptor α agonist (PPT) was administered by intraperitoneal injection at doses of 0.05 gr/kg body weight in corn oil. Treatment started one day before treadmill training and continued throughout the training period. Daily injections were given after running to avoid interference with exercise performance. Controls receive equivalent injections of corn oil.

### Experimental design

Middle-aged female mice were randomly divided into the following experimental groups: 1) sedentary control, 2) sedentary control + corn oil, 3) sedentary + PPT (an ERα agonist), 4) exercised control, 5) exercised + corn oil, and 6) exercised + PPT. A subset of these groups (Protocol 1, see Supplemental Figure 1C) underwent several tests to determine resilience to stress and anxiety-like behavior. Specifically, animals were submitted to the forced swim test to determine baseline “depressive-like” behavior. Next, after habituation to the treadmill procedure, mice were submitted to running exercise for two weeks and then to the elevated plus maze followed by a tail suspension test. Another subset (Protocol 2) was submitted to running exercise for 2 weeks and then sacrificed. Their hippocampi and serum were collected to determine gene expression and protein levels. IGF-I levels in hippocampus were determined in all experimental groups whereas clusterin, Plxna4, and serum IGF-I were determined only in groups 1 and 4. In Group 1 we also evaluated serum E2 levels by ELISA to compare them with a young cohort of young female mice.

### Treadmill running

Mice were subjected to treadmill running for 2 weeks (5 days/week). Mice were familiarized with the treadmill apparatus (Letica, Italy) to minimize novelty stress and then divided in two groups: exercised and non-exercised. The electrical shock system that encourages the animals to run was disconnected to avoid pain stress. The exercise group ran for 40 min at 12m/min, whereas the control group remained for the same time in the treadmill without running. We chose this mild intensity exercise regime to avoid changes in stress hormones [31] that could interfere with post-exercise behavioral assessment. For biochemical assays, a subset of mice was deeply anesthetized and sacrificed right after the last running session. Trunk blood samples were obtained and brains were perfused with 0.9% saline solution and were either snap frozen for ELISA, Western blot and qPCR or further perfused with 4% paraformaldehyde for immunostaining. Additional groups of animals were used for behavioral testing at different times after running (see Fig 1A).

### Elevated plus maze

To assess anxiety-like behavior, animals were placed in the center of an elevated plus maze for 5 min, as described [32]. The maze was at 40 cm from the floor with two opposing protected (closed) arms of 30 cm (length) x 5 cm (wide) x 15.25 (height), and two opposing unprotected (open) arms (30x5x0). Time in the open and closed arms, as well as the number of entries in each arm, were recorded with an automated video-tracking system (Video Tracking Plus Maze Mouse; Med Associates, USA).

### Stress resilience

To determine the effects of exercise on resilience to stress, we submitted mice to two different stressful tests, one before and the other after exercise. Thus, three days prior to treadmill training mice were exposed once to forced swimming as a stressor (Supplemental Figure 1C). One day after completion of exercise training, the tail suspension test (TST) was used to measure coping behavior [33]. We used this second type of stressful test to avoid re-exposure of mice to the first stressor (forced swim) and in this way eliminate any potentially confounding learning component. In the forced swim test (FST), mice were placed in a glass cylinder (12 cm diameter, 29 cm height) filled with water (23°C) to a height of 15 cm (to avoid climbing). The duration of the test was 6 min, with the last 4 minutes scored by a blind observer. The animals keep swimming until they give up or they alternated between swimming and floating. In the TST, mice were individually suspended by the tail from a plastic cage (21x26x15) using adhesive tape (distance from tip of tail was 2 cm); the distance from the floor was 35 cm. Animals struggled to get to the floor until they give up and struggled less frequently. A 6 min test session was videotaped and the time spent immobile was scored by a blind observer and referred as percent of total time of duration of the test. In both tests, animals that keep moving for a longer period of time are considered to have a stronger coping strategy

### Immunoassays

Western blot (WB) and co-immunoprecipitation (co-IP) assays were performed as described before [34]. Actin was used as a loading control for WB, whereas co-IPs were normalized with the antibody used to pull down. Non-immune serum (NIS) was used as a negative control for IPs while total cells lysate as a positive control. See Supplementary Table 1 for antibodies used. IGF-I in serum and tissues was determined using a species-specific ELISA (R&D Systems, USA), as described in detail elsewhere [35]. E2 was evaluated in serum by ELISA (Enzo Life Sciences, USA), following the manufacturer´s instructions. Blood was collected from the heart after pentobarbital anesthesia before trans-cardiac perfusion.

### Endothelial cell cultures

Endothelial cells were obtained as follows: female mice (2 or 9 month-old) were euthanized by C0_2_ exposure; the head was removed, immersed in 70% ethanol and transferred to a Petri dish with cool sterile phosphate-buffered saline (PBS; pH 7.4). The brain was removed under sterile conditions and stored on ice with DMEM/F12+10% FBS+penicillin/antimycotic until dissected. Dissection was carried out in cool sterile PBS (pH 7.4) as follows: meninges were removed and discarded; the clean brain tissue was placed on ice in DMEM /F12+10% FBS+ penicillin/antimycotic, cut with a scalpel into small pieces (for no longer than 1 min), and the pieces transferred to a 50 ml Falcon tube with 25 ml of enzymatic medium. The enzymatic medium (previously filtered with a 0.2 μm membrane) consisted of 45000 units of collagenase II (Gibco 17101-015) diluted in 25 ml of PBS + 1% BSA and 25 μl of 1M CaCl2 and 25 μl of 1M MgCl2. Pieces were incubated 2h at 37 ^0^C and then passed through a 20G needle 3-4 times. The resulting mixture was pipetted through a 70 μm disposable cell strainer into a fresh 50 ml tube. Cells were centrifuged at 1300 rpm for 5 min at 4^0^C, the supernatant aspirated and the pellet re-suspended in 25 ml of sterile PBS + 0.1% BSA and again centrifuged at 1300 rpm for 5 min at 4^0^C. The resulting pellet was re-suspended in 1 ml sterile PBS + 0.1% BSA and human endothelial medium plus 20% FBS, 1 μg/ml hydrocortisone, 4 μg/ml puromycin and antibiotic/antimycotic were added. Cells were plated in plates coated with collagen IV/fibronectin and washed with PBS before seeding. The next day, medium was aspirated, cells washed with PBS and the medium replaced with growth medium (DMEM/F12 plus 20% FBS, 1 μg/ml hydrocortisone, 2 ng/ml bFGF, 0.1 mg/ml Cow Brain Extract and penicillin/antimycotic). Medium was then replaced every 2 days.

### Biotinylated IGF-I uptake

Endothelial cells obtained from 2 or 9-month old female mice were washed with PBS and serum-free DMEM/F12 without red phenol was added. Three hours later, either 1 nM 17β-estradiol (Sigma, USA), 1 nM PPT (Tocris,USA), or 1 nM DPN, an ERβ agonist (Tocris, USA) were added (or equivalent dose of DMSO in control wells), and 2 hours later biotinylated IGF-I (bIGF-I, IbmH, Germany, 0.2 µg/ml) was added. One hour later, cells were lysed and processed for WB.

### qPCR

RNA from endothelial cells was extracted with TRIZOL (Life Technologies, USA) following the manufacturer’s protocol using TaqMan probes. Reactions were performed in an ABI PRISM® 7000 Sequence Detection System. The mRNA levels were normalized with 18sRNA. Primer sequences are shown in Supplementary Table 2.

### Statistical analysis

Statistical analysis was performed using GraphPad Prism 5 software (San Diego, CA, USA). All results are shown as mean ± s.e.m. After testing for normal distribution, for single comparisons we used Student’s *t*-test and for multiple comparisons one or two-way analysis of variance plus Bonferroni’s test. Probability values <0.05 were considered significant.

## Supplementary Material

Supplementary Figure

Supplementary Tables
