# Supplementary material for: Loss of the interaction between estradiol and insulin-like growth factor I in brain endothelial cells associates to changes in mood homeostasis during peri-menopause in mice
Source: Aging (Albany NY). 2019 Jan 11;11(1):174–84. doi: 10.18632/aging.101739 (PMC6339786; doi:10.18632/aging.101739)
Supplement: Supplementary Figure [file aging-11-101739-s001.pdf]

SUPPLEMENTARY FIGURE

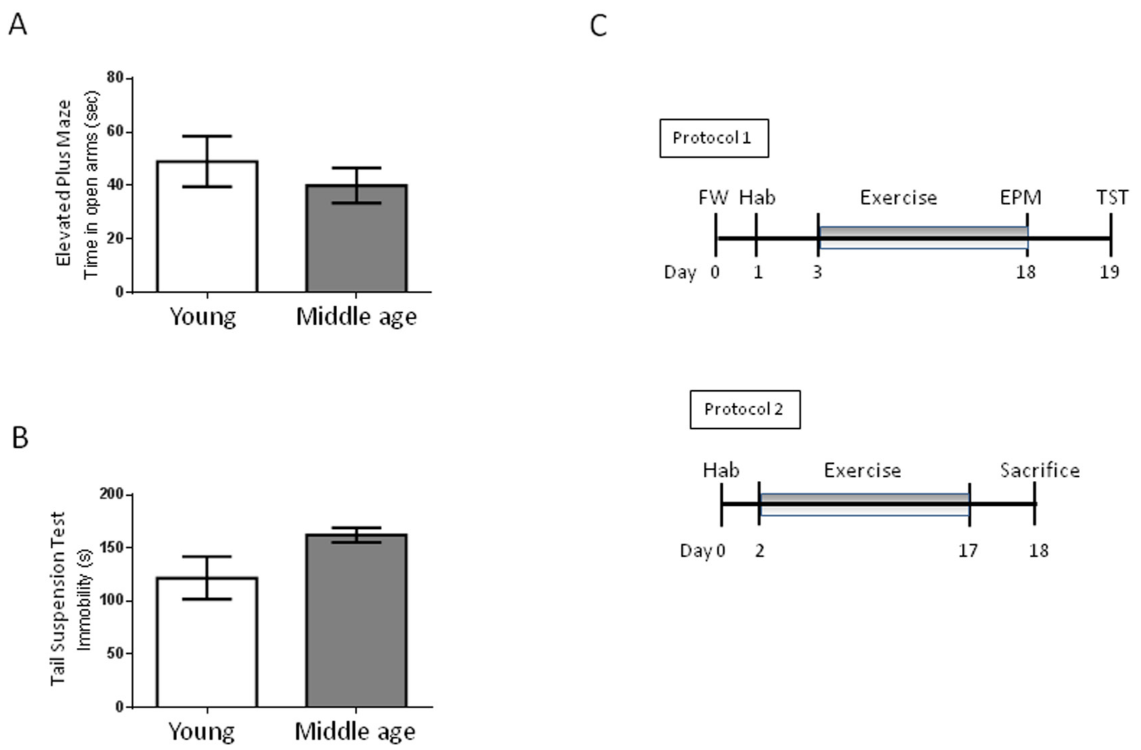

Supplementary Figure 1. (**A**) No differences in anxiety levels were found between young (2 months) and middle-aged (9 months) female mice, as determined by time spent in the open arms of the elevated plus maze. (**B**) No differences were observed either between young and middle-aged females in resilience to stress, as measured by time of immobility in the tail suspension test administered after forced swim. (**C**) Experimental design used in middle-aged female mice. Time line of the 2 experimental protocols used in the study. Hab: habituation to treadmill running; FW: forced swim; EPM: elevated plus maze; TST: tail suspension test.
