# Supplementary material for: Loss of the interaction between estradiol and insulin-like growth factor I in brain endothelial cells associates to changes in mood homeostasis during peri-menopause in mice
Source: Aging (Albany NY). 2019 Jan 11;11(1):174–84. doi: 10.18632/aging.101739 (PMC6339786; doi:10.18632/aging.101739)
Supplement: Supplementary Tables [file aging-11-101739-s002.pdf]

## SUPPLEMENTARY TABLES

**Supplementary Table 1. Antibodies used.**

| Atibody | Reference | Dilution | References | Company |
|---|---|---|---|---|
| IGF-IRβ | Chong, Q. et al 2017 | 1:1000 | Over 30 citations | Cell signalling |
| IGF-IRβ | Pagesy, P. et al. 2016. | 1:200 | Over 190 citations | Santa Cruz Biotechnology |
| ERα | Choi, HS. et al. 2017 | 1:500 | Over 110 citations | Santa Cruz Biotehcnology |
| β-Actin | Shuang Qiu et. Al 2014 | 1:2000 | Over 900 citations | Sigma |

**Supplementary Table 2. Sequence of primers used in qPCR assays.**

| Gene | Specie | Sequence | Reference |
|---|---|---|---|
| IGF-I | Mus musculus | GCTTTTACTTCAACAAGCCCACAGG | Mm00439560_m1 |
| IGF-IR | Mus musculus | GGCCAGAAGTGGAGCAGAATAATCT | Mm00802831_m1 |
| Esr1 | Mus musculus | ACAAGGCTCC CTGCTCAGCAGCCTTG | Mm00433149_ml |
| 18s | Mammalia | | 4319413E |
